# From COPD epidemiology to studies of pathophysiological disease mechanisms: challenges with regard to study design and recruitment process: Respiratory and Cardiovascular Effects in COPD (KOLIN)

**DOI:** 10.1080/20018525.2017.1415095

**Published:** 2017-12-17

**Authors:** Anne Lindberg, Robert Linder, Helena Backman, Jonas Eriksson Ström, Andreas Frølich, Ulf Nilsson, Eva Rönmark, Viktor Johansson Strandkvist, Annelie F. Behndig, Anders Blomberg

**Affiliations:** ^a^ Department of Public Health and Clinical Medicine, Division of Medicine, Umeå University, Umeå, Sweden; ^b^ Department of Public Health and Clinical Medicine, Division of Occupational and Environmental Medicine, Umeå University, Umeå, Sweden; ^c^ Department of Health Science, Division of Health and Rehabilitation, Luleå University of Technology, Luleå, Sweden

**Keywords:** Chronic obstructive pulmonary disease, disease mechanisms, lung function decline, smoking habits

## Abstract

**Background**: Chronic obstructive pulmonary disease (COPD) is a largely underdiagnosed disease including several phenotypes. In this report, the design of a study intending to evaluate the pathophysiological mechanism in COPD in relation to the specific phenotypes non-rapid and rapid decline in lung function is described together with the recruitment process of the study population derived from a population based study.

**Method**: The OLIN COPD study includes a population-based COPD cohort and referents without COPD identified in 2002–04 (*n* = 1986), and thereafter followed annually since 2005. Lung function decline was estimated from baseline in 2002–2004 to 2010 (first recruitment phase) or to 2012/2013 (second recruitment phase). Individuals who met the predefined criteria for the following four groups were identified; group A) COPD grade 2–3 with rapid decline in FEV_1_ and group B) COPD grade 2–3 without rapid decline in FEV_1_ (≥60 and ≤30 ml/year, respectively), group C) ever-smokers, and group D) non-smokers with normal lung function. Groups A–C included ever-smokers with >10 pack years. The intention was to recruit 15 subjects in each of the groups A-D.

**Results**: From the database groups A–D were identified; group A *n* = 37, group B *n* = 29, group C *n* = 41, and group D *n* = 55. Fifteen subjects were recruited from groups C and D, while this goal was not reached in the groups A (*n* = 12) and B (*n* = 10). The most common reasons for excluding individuals identified as A or B were comorbidities contraindicating bronchoscopy, or inflammatory diseases/immune suppressive medication expected to affect the outcome.

**Conclusion**: The study is expected to generate important results regarding pathophysiological mechanisms associated with rate of decline in lung function among subjects with COPD and the in-detail described recruitment process, including reasons for non-participation, is a strength when interpreting the results in forthcoming studies.

## Background

Chronic obstructive pulmonary disease (COPD) is a common disease strongly associated with increasing age and environmental exposure, most commonly own tobacco smoke exposure [1] but globally also indoor exposure to biofuel combustion exhaust [2]. The prevalence of COPD is generally reported in the range of 8–10% among adults. However, actions for smoking control in the society have been followed by reduced smoking in many countries, and a recently published study indicates that the prevalence of COPD has decreased in parallel with changes in smoking habits in Sweden [3]. Still, the underdiagnosis is substantial; merely 20–30% of all individuals with COPD are identified by health care [4,5]. Thus, the results from studies including selected populations recruited from the health care (‘register-based studies’) must be interpreted with care, as the large underdiagnosis will affect generalizability. Still, most studies on COPD epidemiology are cross-sectional, limiting the understanding of the disease process from a general population point of view and, furthermore, seldom include an evaluation of possible pathophysiological mechanisms in relation to disease progress.

Studies with the aim to understand the underlying pathophysiological mechanisms of the disease process in COPD are often limited to small and highly selected study populations [6,7]. Whilst important findings on anti-protease imbalance in COPD have emerged from a number of small selective patient studies, showing negative associations between Matrix MetalloProteinase-9 (MMP-9) and lung function [6,7], such study populations can hardly be considered representative for COPD in the general population; hence, it can be questioned to what extent the observed results are generalizable. However, there are a few population-based studies reflecting similar results; higher plasma metalloproteinase-9 levels, indicating protease-antiprotease imbalance, were associated with lower FEV_1_ percent predicted among subjects in a population-based COPD cohort, when compared to subject without airway obstruction [8]. Nevertheless, to increase the understanding of the pathophysiological mechanisms related to disease progression in COPD, further studies of representative samples from the general population are warranted.

COPD was previously described as a smoke-induced lung injury but is today regarded as a heterogeneous syndrome, comprising several phenotypes [9], which may be related to different pathophysiological mechanisms. Rate of decline in lung function, degree of emphysema, exacerbation frequency, cardiovascular co-morbidity, and tobacco smoke exposure are just a few possible clinical factors related to underlying pathophysiological mechanisms and, thus, involved in the disease process.

The intention of this report is to describe the recruitment process of a study sample from a population-based COPD cohort to a study aiming at evaluating the pathophysiological mechanism in COPD in relation to one of the above-mentioned clinical phenotypes, characterized by rate of decline in lung function. The study was designed to address the hypothesis that certain biomarkers would differ between COPD subjects with rapid decline in lung function compared to those with a non-rapid decline, also including comparisons with subjects with normal lung function, both non-smokers and those with a history of smoking. Here, the recruitment process will be presented, along with rate and reason for non-participation, as well as the basic characteristics of the study population at recruitment.

## Material and method

In 2002–2004, previously examined subjects from four population-based adult cohorts from the OLIN (Obstructive Lung disease In Northern Sweden) studies were invited to re-examination. All subjects fulfilling the spirometric criteria for airway obstruction, FEV_1_/VC <0.70, were identified (*n* = 993) together with age- and sex-matched subjects without obstructive lung function impairment. Since 2005, the study population (*n* = 1986) has been invited to annual examinations with a basic program including spirometry and a structured interview [10]. The current report is based on data from baseline in 2002–2004 and at follow-up in 2010 (first recruitment phase) or 2012/2013 (second recruitment phase).

The study sample in the current report was identified based on predefined criteria, into groups labelled A–D, as defined later. The aim was to include 15 subjects in each of the groups A–D in the study. The intention of groups A and B was to clearly separate COPD with rapid decline in FEV_1_ (A) from COPD with more stable, FEV_1_, i.e. non-rapid decline (B), thus the predefined levels of FEV_1_ decline in these groups were separated by an empty interval. The Global Initiative for Obstructive Lung Disease (GOLD) spirometric criterion for COPD was used [11].

### Predefined groups

Group A –: COPD, rapid decline: COPD GOLD grade 2-3 with a rapid decline in FEV_1_, ≥60 ml/year Ever-smokers with a smoking history of >10 pack years at baseline.
Group B –: COPD, non-rapid decline: COPD GOLD grade 2-3 with a non-rapid decline in FEV_1_, ≤30 ml/year Ever-smokers with a smoking history of >10 pack years at baseline.
Group C –: Ever-smokers with normal lung function: FEV_1_/VC ≥ 70% and FEV_1_ ≥80% of predicted at baseline and at recruitment. A decline in FEV_1_ <20 ml/year. Ever-smokers with a smoking history of >10 pack years at baseline.
Group D –: Non-smokers with normal lung function: FEV_1_/VC ≥ 70% and FEV_1_ ≥80% of predicted at baseline and at recruitment. A decline in FEV_1_ <20 ml/year. Non-smoker at baseline and at recruitment.

The Regional Ethical Review Board at Umeå University, Sweden, approved the study. All participants signed a written informed consent and the study was carried out according to the Helsinki declaration. The study is referred to as ‘Respiratory and Cardiovascular Effects in COPD (KOLIN)’, ClinicalTrials.gov Identifier: NCT02729220.

### Definitions

Smoking habits were classified as non-smoker (less than 1 cigarette per day during a maximum of 1 year), ex-smoker (stopped smoking since at least 12 months), and current smoker (current smoker or stopped smoking within the last 12 months). Ever-smoker was defined as ex-smoker or current smoker. Pack-years at baseline (2002–2004) were calculated.

### Spirometry and spirometric classification

Spirometry was performed using a dry volume spirometer, the Mijnhardt Vicatest 5, the Netherlands following the American Thoracic Society/European Respiratory Society (ATS/ERS) guidelines [12]. Vital capacity (VC) was defined as the highest value of forced vital capacity (FVC), or slow vital capacity (SVC). Reversibility testing was performed if FEV_1_/VC < 0.70 or forced expiratory volume in one second, FEV_1_ < 80 percent of predicted. COPD was defined by the spirometric criteria FEV_1_/VC < 0.70, using the highest value pre- or post-bronchodilation. Disease severity was classified according to the GOLD guidelines [11]; grade 2–3 includes subjects with FEV_1_ < 80 and ≥30 percent of predicted value. Swedish spirometric reference values for FEV_1_ were used [13], corresponding well to FEV_1_ in a symptom-free population of Northern Sweden [14]. Decline in FEV_1_ (ml/year) was calculated as (FEV_1_ at baseline – FEV_1_ at recruitment)/number of years (based on person-days) of follow-up based on highest value pre- or post-bronchodilation.

### Exclusion criteria for participation

Systemic disease and/or treatment with immune-modulating therapy.

Clinical signs of upper or lower respiratory tract infection within the last six weeks.

Asthma

Contraindication for bronchoscopy

– Severe or unstable cardiovascular disease

– Other significant diseases, for example dementia, porphyria, cancer, respiratory insufficiency

– Abnormal pulmonary x-ray prompting specific investigation

### Recruitment procedure

From the OLIN COPD study database, all subjects fulfilling the A–D predefined criteria of lung function, FEV_1_ decline, and smoking history were identified. The predefined criteria were based on baseline characteristics (*n* = 1986) and data at the examinations in 2010 (first recruitment phase) or 2012/2013 (second recruitment phase). The reason for two recruitment phases was to increase the study population, as the first recruitment phase did not identify enough number of subjects in group A and B who met the predefined criteria and no exclusion criteria for participation in the study program. Identified subjects within each of the groups A–D were contacted by telephone in a consecutive order with brief information about the study. Those with a primary interest to participate received written information to their home address and were offered a first appointment with a physician and a research assistant for informed consent, clinical examination, spirometry, and ECG recording. For those who met the study criteria and were willing to participate, the research assistant coordinated a second appointment for the study examinations, including amongst all blood sampling, measurement of arterial stiffness, and bronchoscopy. A pulmonary x-ray was performed in the interval between the first and second appointments. Non-participation was classified, as defined later, at each step of the recruitment procedure.

### Classification of non-participation during the recruitment process

– Fulfilling any of the exclusion criteria

– Declined participation due to unwillingness to undergo the bronchoscopy procedure

– Declined participation due to other reason, as specified

– Impossible to reach by telephone

### Statistics

Descriptive statistics were performed with Statistical Package for the Social Science, version 22.

## Results from the recruitment process

### Basic characteristics of the four groups; A–D at baseline in 2002–2004

Basic characteristics at baseline, in 2002–2004, of all subjects in the study database fulfilling the pre-set criteria regarding lung function, FEV_1_ decline, and smoking history for groups A–D are presented in Table 1. Group A included 37 subjects (28 male, 9 female), group B 29 subjects (19 male, 10 female), group C 41 subjects (16 male, 25 female), and group D 55 subjects (37 male, 18 female).

**Table 1.** Total number of subjects within the groups A–D identified in the OLIN COPD study data base at baseline in 2002–2004 and follow-up in 2010 (first recruitment phase) or 2012/2013 (second recruitment phase); characteristics at baseline in 2002–2004 for each of the groups.

	A (*N* = 37)	B (*N* = 29)	C (*N* = 41)	D (*N* = 55)
Age, mean (SD)	57 (7)	59 (6)	56 (7)	57 (7)
Age, range	47–67	48–67	37–68	41–67
BMI, mean (SD)	25.7 (3.5)	26.8 (4.2)	26.0 (3.1)	27.9 (5.1)
Women, *n* (%)	9 (24.3)	10 (34.5)	18 (43.9)	25 (45.5)
Ex-smoker, *n* (%)	9 (24.3)	18 (62.1)	29 (70.7)	0
Current smoker, *n* (%)	28 (75.7)	11 (37.9)	12 (29.3)	0
Pack years, mean (SD)	27 (11.4)	26 (18.3)	22 (12.0)	0
FEV_1_% pred^a^, mean (SD)	77.3 (14.6)	61.5 (13.5)	92.3 (11.0)	93.8 (16.0)
Prod cough, *n* (%)	24 (64.9)	15 (51.7)	6 (14.6)	14 (25.9)
mMRC ≥ 2, *n* (%)	11 (30.7)	27 (93.1)	0	10 (18.2)
Exacerbations^b^	10 (27.0)	12 (41.4)	4 (9.8)	10 (18.2)
Heart disease, *n* (%)	8 (21.6)	7 (24.1)	9 (22.0)	4 (7.3)
Diabetes, *n* (%)	0	2 (6.9)	5 (12.2)	3 (5.5)

^a^Based on values best of pre- and post-bronchodilation.

^b^Defined as contact with health care during the last 12 months due to respiratory problems.

### Non-participation

Groups C and D, but not groups A and B, reached the preset aim of 15 participants; group A included 12 participants and group B 10 participants after inclusion of two individuals with a FEV_1_ decline of 33 ml/year (thereby exceeding the preset criteria ≤30 ml/year). The reasons for non-participation in the study program among subjects fulfilling the pre-set criteria for group A and B, respectively, are shown in Table 2. In group A, 17 out of 37 subjects, 45.9%, were not eligible due to exclusion criteria (*n* = 15) or death (*n* = 2), while in group B, 10 out of 29 subjects, 34.4%, were not eligible due to exclusion criteria (*n* = 9) or death (*n* = 1). In group A, 5 out of 37 individuals, 13.5%, declined bronchoscopy, and in group B, 6 out of 29 individuals, 20.7%, declined bronchoscopy.

**Table 2.** The reason for non-participation in the study program among subjects classified as groups A and B, respectively.

	Group A	Group B
Non-participants/total group	25/37	19/29
Deceased	2	1
Exclusion criteria	15	9
– Significant cardiovascular disease	3	1
– Other significant diseases	8^a^	2
– Immunosuppression	4	2^b^
– History of asthma	-	*4*
Declined bronchoscopy	5	6
Social reason	2	3
Not possible to reach by phone	1	1

^a^One due to accidently found lung tumor in chest x-ray.

^b^One due to lung transplantation.

### Characteristics of study participants at recruitment in 2010 respectively 2012/2013

At baseline in 2002–2004, all subjects participating (_p_) in the study program, groups A_p_–C_p,_ had a smoking history of at least 10 pack-years. In COPD with rapid decline (group A_p_), there were 9 active smokers and 3 ex-smokers, and in non-rapid decline (group B_p_), there were 3 active smokers and 7 ex-smokers. In group C_p_, there were 3 active smokers and 12 ex-smokers. Characteristics of the participants in groups A_p_–D_p_ at the examination in 2010 (first recruitment phase) respectively 2012/2013 (second recruitment phase) are shown in Table 3. The absolute numbers of current smokers had decreased within groups A_p_–C_p_ since baseline.

**Table 3.** Characteristics at the time for the examination in 2010 (first recruitment phase) respectively 2012/2013 (second recruitment phase) of the participants in groups A_p –_ D_p._

	A_p_ (*N* = 12)	B_p_ (*N* = 10)	C_p_ (*N* = 15)	D_p_ (*N* = 15)
Age, mean (SD)	61 (6)	67 (6)	64 (7)	63 (8)
BMI, mean (SD)	26.4 (3.9)	25.6 (2.8)	26.4 (2.1)	28.2 (4.4)
Sex; female/male	2/10	4/6	8/7	4/11
Current smoker/ex-smoker	9/3	3/7	4/11	0/0
FEV_1_/VC^a^, mean (SD)	0.50 (0.11)	0.54 (0.08)	0.76 (0.02)	0.78 (0.04)
FEV_1_% predicted^a^, mean (SD)	65.8 (14.1)	65.4 (12.0)	107.3 (13.9)	106.6 (14.6)
Range	33.0–78.4	45.5–79.2	86.4–135.2	84.3–136.7
FEV_1_ decline ml/year, mean (SD)	−87.3 (25.2)	−10.1 (20.3)	−1.0 (11.2)	−5.3 (14.0)
Physician-diagnosed COPD	5 (41.7)	8 (80.0)	0	0
Productive cough, *n* (%)	3 (25.0)	4 (40.0)	2 (13.3)	1 (6.7)
mMRC ≥ 2, *n* (%)	2 (16.7)	4 (44.4)^d^	0^d^	0
Exacerbation mild^b^, *n* (%)	1 (8.3)	2 [20]	0	0
Exacerbation moderate^c^, *n* (%)	0	2 (20.0) ^e^	1 (6.7) ^f^	0
Heart disease^g^, *n* (%)	3 (25.0)	3 (30.0)	3 (20.0)	2 (13.3)
Diabetes, n (%)	2 (16.7)	2 (20.0)	1 (6.7)	1 (6.7)

^a^Based on values best of pre- and post-bronchodilation.

^b^Increased medication or received new medication during the last 12 months.

^c^Treatmed with antibiotics and/or oral steroids during the last 12 months.

^d^One person in each of the groups B and C was excluded due to impaired mobility for reasons other than respiratory.

^e^Two persons were treated, one of them was treated twice.

^f^Antibiotics.

^g^Heart disease include any of angina pectoris, percutaneous coronary intervention (PCI), coronary artery bypass surgery (CABG), myocardial infarction, or chronic heart failure.

## Discussion

Tobacco smoking is the most well-known risk factor for COPD. Some smokers with COPD experience rapid decline in lung function, while others have low or ‘normal’ rate of decline and, at the same time, there are smokers with normal lung function without enhanced rate of lung function decline, similar to non-smoking subjects. In this report, we describe the recruitment process of a study sample and the design of a study with the aim to increase the understanding of underlying pathophysiological mechanisms in COPD contributing to differences in lung function decline, non-rapid and rapid decline in FEV_1_. The study sample was recruited from the OLIN COPD study, including a well-characterized large population-based COPD cohort and age- and sex-matched referents without obstructive lung function impairment. The OLIN COPD study includes annual follow-ups since recruitment in 2002–2004, and has so far contributed with data covering a wide area as the following examples show; from genetics, disease mechanisms and mortality to comorbidities, physical activity, muscle strength, fatigue, and health economics, including also evaluation of prognostic factors [8,15–24]. The study provides valid data for estimation of lung function decline among subjects both with and without COPD [10]. The recruitment of subjects without COPD reached the set goal, while this was not the case for those with COPD, and among them, the most common reasons for a fairly high exclusion rate were medical conditions contraindicating bronchoscopy or inflammatory conditions/medication expected to affect the outcome.

It has been suggested that a decline in FEV_1_ among subjects with COPD needs to be evaluated across several years, as lung function values may naturally fluctuate somewhat between repeated examinations performed at shorter time intervals [25]. In the present study, we included an observation period of at least six and even up to 10 years as a basis for calculating decline. Still, the chosen levels of defining rate of decline in FEV_1_ may be discussed. Normal rate of decline in FEV_1_ among middle-aged adults is described as 25–30 ml/year [26]. Here, we chose an even stricter criterion for groups C and D, having normal lung function at recruitment and follow-up as well as an annual decline in FEV_1_ within the range of <20 ml. The two groups with COPD were defined to discriminate between subjects with rapid decline in FEV_1_ (A) and subjects without rapid decline in FEV_1_ (B). There is, however, no established definition of rapid decline in lung function, or specifically rapid decline in FEV_1_, in the literature. In 1977, Fletcher and Peto published the classic illustration of lung function decline in relation to smoking habits over eight years among 792 men [27], and they also discussed 60 ml/year as a cut of for rapid decline. More recent data from the COPD Gene study showed that the overall mean (SD) annual decline among subjects with GOLD 2 was 45.6 (61.1) ml/year across a 5-year observation time [28] and, in another study, the mean rate of decline among incident cases of COPD was 51 ml/year, estimated throughout a 10-year period [29]. To exemplify various cut-offs for rapid decline, approximately one third of the patients in a hospital-based COPD cohort were classified as having a rapid decline in FEV_1_ with a mean decline of 78 ml/year (95%CI 73–83) [30], while in a population-based study, half of the subjects above the age of 40 were identified as rapid decliners with a mean (SD) decline in FEV_1_ of 53 (21) ml per year [31]. In these two referred studies, the rest of those with COPD, i.e. non-rapid decliners, had a mean decline in FEV_1_ of 26 ml (95%CI 23–29 ml) and 27 (18) ml per year, respectively. For comparison, in a study including patients from pulmonary clinics, rapid decline was set at 40 ml/year [32], based on the findings in the ECLIPSE study [33]. Our decision to employ 60 ml/year as a cutoff for group A, representing a more rapid decline in FEV_1_, and <30 ml/year, as a more normal rate of decline in FEV_1_ for group B, can be considered well-motivated and in line with the above-referred publications.

In the OLIN COPD cohort, group D, non-smokers with normal lung function and low rate of decline in FEV_1_, was as expected the largest group, whereas group B, COPD with a smoking burden of at least 10 pack-years and a fairly normal rate of decline in FEV_1_, was the smallest group. For comparison, in the previously referred study of incident cases of COPD, just over a quarter of the incident cases, 27.6%, had a decline of less than 30 ml/year [29] and, on the other hand, in the ECLIPSE-study, only 38% of the participants presented a decline in FEV_1_ above 40 ml/year during the three-year follow-up [33]. Furthermore, the previously referred recent publication in *New England Journal of Medicine* [31] provides additional support for the assumption of a low rate of decline in lung function in a sub-population of individuals with COPD; rapid decline in lung function is not obligate among subjects with COPD and a FEV_1_ below 80% of predicted, corresponding to GOLD 2 and higher. One explanation is that lung function never reached expected normal values in this group and that they by normal rate of decline in lung function eventually fulfilled the spirometric criteria for COPD [31,34], and at least some of those with COPD in group B in our study may belong to such a trajectory.

There were no problems to recruit the intended number of 15 subjects to groups C and D, while this goal could not be reached in COPD groups, group A and B, despite a second recruitment phase. To get closer to the goal of recruitment, we allowed two individuals with a FEV_1_ decline of 33 ml/year to be included in group B. The main reason for non-participation in groups A and B was exclusion due to clinical contraindications for bronchoscopy, or medical conditions, such as inflammatory diseases or need of immunosuppressive drugs, which may affect the results on inflammatory endpoints in the main study; in total, nearly every other subject in group A and more than one out of three in group B. It is well known that co-morbidities are common among subjects with COPD and cardiovascular diseases are the most frequent [17,35]. The reviewing of non-participation revealed that co-morbidities contributed to a considerable obstacle when recruiting participants to this study also including an invasive procedure, such as bronchoscopy. It was evident that, even with access to a large population-based longitudinal COPD cohort, we could not meet the intended number of participants in groups A and B.

Most studies on pathophysiological mechanisms in COPD are cross-sectional and include highly selected study populations unrelated to population-based samples [36,37]. When recruiting participants to groups A and B in the current study, non-participation was fairly high among subjects with COPD. Through detailed data from the recruitment process, we will be able to discuss non-participation in relation to forthcoming results and, moreover, generalizability in relation to COPD in the society in a way that most other studies do not allow. However, in a longitudinal study also, the healthy survivor effect must be taken into account. In a previous publication based on cross-sectional data collected in 2010 from the OLIN COPD study, it was reported that subjects deceased from baseline in 2002–2003 until 31 December 2009 were older and had a higher prevalence of COPD, productive cough, and heart disease compared to the participants in the 2010 examinations, thus supporting a healthy survivor effect [18]. As a consequence of an expected healthy survivor effect, forthcoming results from the current study may rather underestimate than overestimate differences and/or associations when comparing groups.

In this study, the fixed ratio spirometric criterion for COPD according to GOLD was used. It is well-known that the fixed ratio will overestimate COPD among elderly [38] and the lower limit of normal criteria are nowadays recommended to be used in epidemiological research [39]. Still, most current clinical guidelines for diagnosis and management of COPD are based on the fixed ratio criterion and GOLD stage 2 and above are considered to identify clinically relevant disease [40,41]. Thus, the fixed ratio criterion is still highly clinically relevant for spirometric classification of COPD and allows the results to be interpreted in the clinical setting.

The OLIN longitudinal COPD study that was the basis for recruitment of the present study population includes a large population-based COPD cohort comparable to that of NHANES I [42]. Long-term follow-ups of population-based COPD cohorts, in which COPD is classified according to spirometric criteria of accepted guidelines, are rare. The Copenhagen City Heart study was recruited in 1976 and included more than 2000 subjects classified as COPD and, so far, there are three follow-ups within 25 years, the latest in 2001–2003 [43]. However, it is an open study including new subjects at each of the follow-ups and, thus, not comparable to either the OLIN COPD or the NHANES studies. In a recent publication from the Copenhagen City Heart, including data from 8000 subjects with a follow-up of approximately 18 years, merely 303 cases of COPD with at least two lung function tests were identified, and their mean (SD) decline in FEV_1_ was 46 (28) ml/year [44]. There are few population-based longitudinal studies with several years of follow-up providing large enough COPD cohorts to study pathophysiological mechanisms in relation to disease progression, here assessed as rate of decline in FEV_1_.

### Conclusion

The presented study design provides a good basis for evaluating underlying pathophysiological mechanisms contributing to differences in lung function decline, rapid and non-rapid decline, among subjects with COPD derived from a population-based sample, also including comparison with ever-smokers and non-smokers with normal lung function. A large burden of comorbidities among subjects with COPD was the most important factor affecting participation in this study including an intervention procedure, such as bronchoscopy. When aiming at recruiting patients with a specific COPD phenotype from longitudinal population-based studies, various comorbidities may significantly disable the recruitment process, even if the basis for recruitment is considerably large. However, our study is expected to generate important results regarding pathophysiological mechanisms related to disease progress, assessed as lung function decline, among subjects with COPD, and the in-detail described recruitment process, including reasons for non-participation, is a strength when interpreting as well as assessing the generalizability of the results.
